# Imaginal Disc Growth Factor 6 (Idgf6) Is Involved in Larval and Adult Wing Development in *Bactrocera correcta* (Bezzi) (Diptera: Tephritidae)

**DOI:** 10.3389/fgene.2020.00451

**Published:** 2020-05-06

**Authors:** Yan Zhao, Zhihong Li, Xinyue Gu, Yun Su, Lijun Liu

**Affiliations:** Department of Entomology, College of Plant Protection, China Agricultural University, Beijing, China

**Keywords:** *Bactrocera correcta*, imaginal disk growth factor 6, RNA interference, death, wing malformation

## Abstract

In insects, imaginal disk growth factors (IDGFs), an important component of the glycoside hydrolase 18 (GH18) family of chitinases, have been reported to be associated with the maintenance of the cuticle and molting. However, there is little knowledge of their function. In this study, imaginal disk growth factor 6 (*Idgf6*), which is an *Idgf*, was first identified and cloned from the guava fruit fly *Bactrocera correcta* (Bezzi) (Diptera: Tephritidae), one of the most serious pest insects in South China and surrounding Southeast Asian countries. This gene encodes IDGF6 protein with a conserved domain similar to ChiA chitinases, the glycoside hydrolase 18 (GH18) family of chitinases, according to NCBI BLAST. Phylogenetic analysis indicated that all *Idgf6s* were highly conserved among similar species. Subsequent temporal expression profiling revealed that *Idgf6* was highly expressed in both the late-pupal and mid-adult stages, suggesting that this gene plays a predominant role in pupal and adult development. Furthermore, RNA interference experiments against *Idgf6* in *B. correcta*, which led to the specific decrease in *Idgf6* expression, resulted in larval death as well as adult wing malformation. The direct effects of *Idgf6* silencing on *B. correcta* indicated its important role in development, and *Idgf6* might be further exploited as a novel insecticide target in the context of pest management.

## Introduction

The epithelial apical extracellular matrix (ECM) is a specialized structure comprising secreted or transmembrane fibrous proteins and polysaccharides, whose composition varies widely, from chitinaceous cuticles of insects to cellulose in plants (Özturk-Çolak et al., 2016; Vuong-Brender et al., 2017; Cosgrove, 2005). Cuticle of insects is an exoskeleton covering the body and internal organs as an epithelial surface Exoskeleton is essential for controlling body shape, epithelial barrier formation, and epidermal wound healing and protects cells from direct contact with pathogens, toxins or pesticides (Galko and Krasnow, 2004; Yoshiyama et al., 2006; Moussian and Uv, 2010; Uv and Moussian, 2010; Turner, 2009; Toshio et al., 2010). It also further provides a challenge to maintain homeostasis of body fluids (Jaspers et al., 2014). Moreover, recent work has established an important role of the ECM in shaping various organs, such as *Drosophila* wings (Fernandes et al., 2010). Based on the conservation of amino acid sequences, several conserved motifs and protein folding, chitinase has been divided into two families, named family 18 and family 19 glycosyl hydrolases (Coutinho and Henrissat, 1999; Henrissat, 1999). The glycoside hydrolase 18 (GH18) family, due to its characteristic glycol-18 domain, is a key family in insects that is widely distributed in all kingdoms, including bacteria, plants and animals (Tsai et al., 2001; Zhu Z. et al., 2004; Zhu et al., 2008a; Arakane and Muthukrishnan, 2010; Zhang et al., 2011a; Huang et al., 2012; Hussain and Wilson, 2013). Previous studies have shown that insects utilize multiple GH18 family chitinolytic enzymes for degrading, remodeling and binding to chitin and possibly for chitin synthesis (Zhu Q.S. et al., 2004).

Polypeptide factors with mitotic activity in invertebrates were first reported to be encoded by imaginal disk growth factors (IDGFs), belonging to group V chitinase, which are an important member of the GH18 family (Žurovcová and Ayala, 2002). *Idgfs* were originally identified and isolated from *Drosophila* S2 or imaginal disk cells on conditioned media (Kirkpatrick et al., 1995; Kawamura et al., 1999). In *Drosophila melanogaster*, there are six genes encoding IDGFs including *Idgf1*, *Idgf2*, *Idgf3*, *Idgf4*, *Idgf5*, and *Idgf6* (Kirkpatrick et al., 1995; Kawamura et al., 1999; Bryant, 2001; Varela et al., 2002). According to previous studies, in imaginal disk cell culture, *Idgfs* promote growth, proliferation, cell polarization, and motility (Kawamura et al., 1999). Some IDGFs are required for normal ECM formation, larval and adult molting or innate immune responses and wound healing (Zhang et al., 2011b; Kucerovaa et al., 2016; Pesch et al., 2016; Broz et al., 2017). Some studies have focused on the function of individual *Idgf* genes; for example, by individually knocking down genes in cuticle-secreting tissues, a large number of *Idgfs* have been shown to be involved in cuticle molting during the larval and pupal stages. This result was then supported by gene-specific spatial-temporal expression profiles and by developmental lethality profiles upon gene knockdown. Moreover, after the genes were knocked down, the mutants were highly susceptible to mechanical stresses and bacterial infections (Pesch et al., 2016). The non-enzymatic *Idgfs* play an important role in protecting newly synthesized cuticle matrix from degradation, which can stabilize and expand the size of ECM in larvae (Pesch et al., 2016). The target gene of our study, *Idgf6*, is one of the first identified *Idgf* genes; *Idgf6* was isolated by Kirkpatrick et al. and localized on the second chromosome at 53D (*Idgf6* is synonymous to Cht13 and DmDS47) (Kirkpatrick et al., 1995; Zhang et al., 2011b). Pesch et al. investigated the molecular network in *Idgf6* RNAi-induced mutants and showed that *Idgf6* RNAi-induced mutants exhibited the strongest lethality and most severe cuticle defects among other mutants (Pesch et al., 2016). *Idgf6* is critical for larval cuticle barrier formation and protection against invasive microorganisms and mechanical stresses (Pesch et al., 2016). Overall, few studies of this gene have focused on larval development, and knowledge of this gene in insect pupal and adult development is limited.

The guava fruit fly *Bactrocera correcta* (Bezzi) (Diptera: Tephritidae) is an economically important insect pest that is widely distributed in South China and other surrounding Southeast Asian countries (Liang et al., 1996; Drew and Raghu, 2002). This fruit fly infests a wide variety of types of commercial fruits, including guava, mango and peach, and vegetables in tropical and subtropical regions of the world (Liang et al., 1996; Bezzi, 1916; White and Elsomharris, 1992). Due to its polyphagous nature, along with its highly adaptive, reproductive and dispersal capabilities, it is considered to be a highly invasive fruit pest species that has been listed as a quarantine pest species by many countries and regions (White and Elsomharris, 1992). Therefore, the control of the guava fruit fly is thus increasingly important. Insect cuticle and molting have been the focuses of pest control research; consequently, clarification of insect *Idgf* gene expression should provide new knowledge that is useful for pest control (Togawa et al., 2007). Although *Idgfs* have been studied systematically in model insects such as *D. melanogaster*, relevant information is limited in*B. correcta*.

In the current study, we first cloned and identified the full-length cDNA of *Idgf6* from *B. correcta*, and previously, little was known about *Idgf6* in nonmodel organisms. We then analyzed the temporal expression pattern of *Idgf6* in eight different developmental stages of *B. correcta* using qRT-PCR. RNA interference technology was applied to explore the function of *Idgf6* in *B. correcta* at larval and adult stages. The *Idgf6* gene was found to play an important role in fruit fly development. Silencing of the *Idgf6* gene resulted in larval death and adult wing malformation. Our data reveal a critical role for *Idgf6* in insect development and thus provide new insights into pest management.

## Materials and Methods

### Experimental Insects

The *B. correcta* population used in this study was collected from Yunnan Province and was cultured in the laboratory at 25 ± 0.5 °C with 65 ± 5% relative humidity under a 14 h light/10 h dark photoperiod. All adults were maintained under the same conditions before starting the experiments to ensure the consistency of the experimental materials. The population had been cultured for approximately 10 generations to eliminate the influence of the local environment. The insects were fed artificial diets as previously described (Yuan et al., 2006). Two hundred individuals were maintained in three insect rearing cages (45 cm × 45 cm × 50 cm) in this experiment.

For temporal expression analysis, we collected samples from different stages: 1^st^ instar larvae (2-day-old indicates 2 days post hatching), 3^rd^ early instar larvae (5-day-old), 3^rd^ instar larvae (7-day-old), early pupae (1-day-old indicates 1 day post pupating), medium pupae (5-day-old), late pupae (9-day-old), early adults (1 day post eclosion), and late adults (10 days post eclosion). Each stage had five replicates, and different numbers of individuals at each stage were collected to detect the expression because of different sizes of insects. Fifty individuals for 1st instar larvae, thirty individuals for 3rd instar larvae and all the pupa stages, ten for the adult stages. For the functional study, five replications were performed for each treatment, and each replicate contained 30 larvae. And for the functional study of the larval stage and adult stage, we obtained samples from 3^rd^ instar larvae and 2-day-old adults, respectively. The samples were immersed in an RNA storage reagent (Tiangen, Beijing, China), immediately frozen with liquid nitrogen and stored at −80°C for further experiments.

### Bioinformatics Analysis

#### RNA Extraction, Reverse Transcription, and cDNA Synthesis

RNA was extracted from the whole body using the RNAsimple Total RNA Kit (Tiangen, China) in accordance with the manufacturer’s protocol. The extracted RNA was immediately dissolved in RNase-free water, and then was checked for quality, concentration, and purity using a NanoVue UV–Vis spectrophotometer (GE Healthcare Bio-Sciences, Uppsala, Sweden) at 260 and 280 nm. RNA integrity was checked by 1% agarose gel electrophoresis at 180 V for 16 min. Five biological replicates were conducted per treatment. Finally, first-strand cDNA was synthesized from 1000 ng of total RNA using the PrimeScript@ RT reagent Kit with gDNA Eraser (Perfect Real Time) (Takara, Japan) following the manufacturer’s instructions.

#### ORF Cloning of *B. correcta Idgf6* and Sequence Analysis

To verify the ORF of *Idgf6* in *B. correcta*, primers were designed based on the conserved regions of *Idgf6* in *B. oleae*, *Ceratitis capitata*, and *D. melanogaster* (sequence from GenBank) and the sequence of *Idgf6* from the *B. correcta* transcriptome (No. MK450457). DNAMAN v.6.03 (Lynnon Biosoft, San Ramon, CA, United States) was used for sequence alignment. The primers for cloning are listed in Table 1. The open-reading frame (ORF) sequence of *Idgf6* was amplified using PrimeSTAR high-fidelity DNA polymerase (Takara, Dalian, China) following the manufacturer’s protocol. The PCR products were isolated, purified and ligated into a pGEM-T Easy vector (Promega, Beijing, China) and sequenced by a company (BGI, Beijing, China).

**TABLE 1:** Primers used for cloning, real-time qRT-PCR amplification and dsRNA synthesis.

Gene	Primer	Sequence	Size (bp)
*Bc 18s rRNA-rt*	*18s*-rt-F	5’- GCGAGAGGTGAAATTCTTGG -3’	192
	*18s*-rt-R	5’- CGGGTAAGCGACTGAGAGAG -3’	
*Bc Idgf6-rt*	*Idgf6*-rt-F	5’-CGGACGAGAAGAGCAGC-3’	176
	*Idgf6*-rt-R	5’-GGCACGCAGTATGGGAT-3’	
*Bc cloneIdgf6-1*	*Idgf6*-whole seq-F	5’-GCGTGTATTTGCTTGTTG-3’	1398
	*Idgf6*-whole seq-R	5’-CGCAGTATGGGATATTTATC-3’	
*Bc dsIdgf6*	*Idgf6*-dsRNA-F	5’-AGCTGCCCTTGCGTGTAT-3’	542
	*Idgf6*-dsRNA-R	5’-GAACCATCAGCGCCTTCA-3’	

The ORF and conserved domain were identified with ORF Finder software^1^ and NCBI BLAST results^2^. To predict the conserved domains of *B. correcta Idgf6*, *Idgf6* protein sequences from 23 species in Drosophilidae and Tephritidae were collected by BlastP in GenBank (Supplementary Table S1) and aligned with the sequence of *B. correcta Idgf6* with ClustalX 2 software and GeneDoc 2.7.0 (Nicholas et al., 1997; Larkin et al., 2007).

### Phylogenetic Analysis

The integrity of homologous amino acid sequences of other species was retrieved from the NCBI server. Sequences were first aligned by the conserved sequences using Geneious v10.22 (Kearse et al., 2012), and then phylogenetic analysis was performed using the maximum-likelihood method with RAxML software (Stamatakis, 2014). One thousand bootstrap iterations were conducted to obtain branch support values.

#### Temporal Expression Pattern of *Idgf6* by qRT-PCR

Following first-strand cDNA synthesis of *Idgf6*, qRT-PCR was performed using SYBR^®^ Premix Ex Taq^TM^ II (Tli RNaseH Plus) (Takara, Japan) on an ABI 7500 instrument (United States). The thermocycler conditions were 95°C for 30 s, followed by 40 cycles at 95°C for 5 s and 52°C for 34 s. Melting curve analysis was performed at the end of each expression analysis using the following conditions: 95°C for 15 s, followed by 52°C for 60 s. The sequences of the qRT-PCR primers used for the reference and target genes are described in Table 1. The relative expression level was calculated using the 2^–ΔΔC^*^*T*^* method (Chen and Wagner, 2012), with 18S rRNA as the reference gene (Gu et al., 2019). Fold changes were determined after the relative expression values were standardized using the lowest value.

#### Silencing of *Idgf6* by RNAi

Double-stranded RNA of *Idgf6* (ds*Idgf6*) was used to knock down *Idgf6* expression, and double-stranded RNA of green fluorescent protein (ds*GFP*) was used as the negative control. We synthesized dsRNAs with the T7 RiboMAX Express RNAi system (Promega, United States) using specific primers containing a T7 promotor sequence (Table 1). Then, dsRNA was purified using phenol, chloroform and ethanol, according to the manufacturer’s instructions, and dissolved in RNase-free water.

The 3^rd^ early instar larvae (5-day-old) of *B. correcta* were collected and placed into a 50 ml tube with 3 holes on the lid. Five replications were performed for each treatment, and each replicate contained 30 larvae. Three grams of artificial diet material with 30 μl of a dsRNA solution was used for feeding, and the concentration of the dsRNA solution for the primary exposure was 1000 ng/μl. The larvae were first fed with ds*GFP* and ds*Idgf6* for 48 h, and then transferred to a new artificial diet with the same treatment for another 48 h. After 96 h, the larvae developed to maturity. Larval mortality was counted, and larval body size was measured; 5 larvae were killed for RNAi efficiency detection. The remaining individuals were fed until the adult stage was reached and were used for phenotype observation on the 2^nd^ day after emergence. The mortality of emerged individuals was recorded twenty days after the flies emerged.

### Statistical Analysis

All experiments included five biological replicates. Statistical analysis was performed using SPSS 20 (IBM Corporation, United States). One-way ANOVA followed by Tukey’s HSD tests was applied to gene expression data to test for significant differences among different developmental stages, and the means were separated using a least significant difference test at a significance level of *P* < 0.05. An independent samples *t*-test (*P* < 0.05 and *P* < 0.01) was used to determine the significance of differences between the treatment and control in the dsRNA injection assay. All data are expressed as mean ± standard error (SE).

## Results

### Cloning and Characterization of *Idgf6*

**FIGURE 1:**
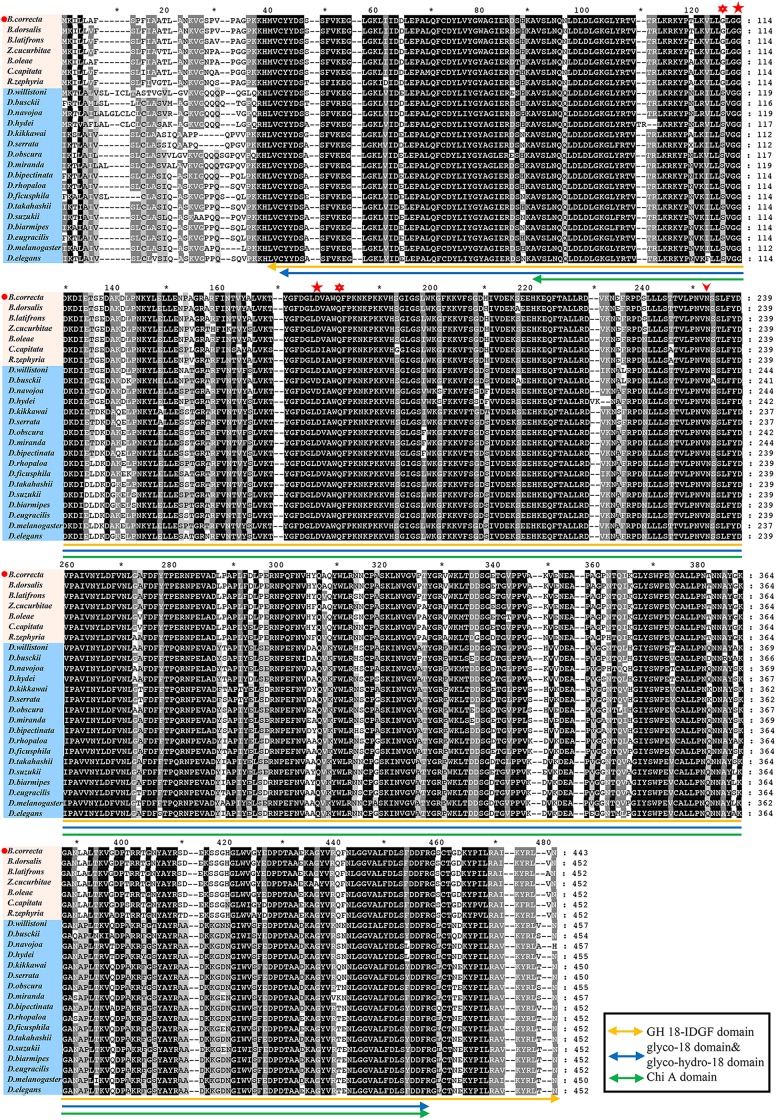
Protein sequence alignments of IDGF6 proteins in Drosophilidae and Tephritidae fruit flies based on NCBI BLAST results. The alignments that present one predicted and conserved domain similar to ChiA chitinases, the glycoside hydrolase 18 (GH18) family of chitinases. Asterisks and star indicate the positions of residues that have been shown to be required for catalytic activity in bacterial chitinase (Watanabe et al., 1993). For the species both in Drosophilidae and Tephritidae, the second and third (star) match the required residues in chitinases, but the fourth (asterisks) is E in chitinases while Q in IDGFs. As for the first (asterisks), in Drosophilidae it is S, which matches the required residues in chitinases, but in Tephritidae it is G. All species IDGF sequences contain single consensus motif (arrowhead) for N-linked glycosylation (Kirkpatrick et al., 1995) that is missing in chitinase.

**FIGURE 2:**
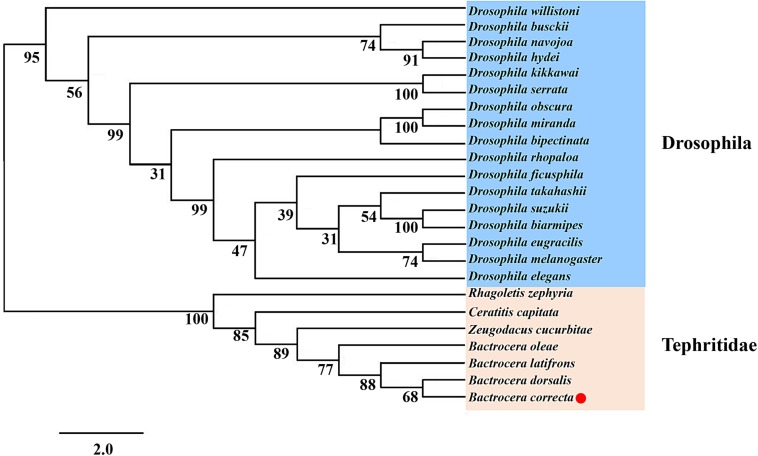
Phylogenetic analysis of *Idgf6* using the maximum-likelihood method in RAxML. One hundred bootstrap iterations were conducted to obtain branch support values. The *B. correcta Idgf6* sequence we obtained is labeled with a red triangle. The amino acid and nucleotide sequences were downloaded from NCBI. The accession numbers of the genes are designated with the corresponding abbreviations and are listed in Supplementary Table S1.

*Idgf6* (GenBank accession no. MK450457) was cloned from *B. correcta*. Five ORFs, which were 1254 bp encoding 465 amino acids, were identified. Preliminary predictions of the conserved domains of the IDGF6 protein with NCBI BLAST showed that one conserved domain similar to ChiA chitinases, the glycoside hydrolase 18 (GH18) family of chitinases was predicted (**Figure 1**). Compared to the *Drosophila* species, which has one amino acid substituent, there are two amino acid substituents in the Tephritid fruit flies, which can eliminate the catalytic activity of chitinase (**Figure 1**). Besides, among all the Tephritid fruit flies, we found that there was one type of gene with the same domain as the phylogenetic tree (Figures 1, 2). Nucleotide sequence analysis revealed that the *Idgf6* of *B. correcta* had the highest identity with a homolog from *B. dorsalis* (96.73%), followed by the *Idgf6* of *B. oleae* (92.82%), *Zeugodacus cucurbitae* (90.67%), and *B. latifrons* (88.52%). Compared to the similar *Drosophila* species, the sequence had the highest identity with *Idgf6* of *D. navojoa* (73.44%).

Using the protein sequences, we analyzed the phylogenetic relationships between *Idgf6* in *B. correcta* and other *Idgf6s* with the maximum-likelihood method. The phylogenetic tree revealed the relationship between insect *Idgf6*s (Figure 2). All *Idgf6*s were highly conserved among similar species. The *Idgf6* in *B. correcta* was clustered close to that in *B. dorsalis*. We clearly observed that *Idgf6s* of the Tephritid fruit flies and the *Drosophila* fruit flies were clustered in two branches of the phylogenetic tree. The amino acid sequence alignment and evolutionary relationship suggested that *Idgf6s* were highly conserved among the Tephritid species.

### Expression of *Idgf6* in Eight Different Developmental Stages of *B. correcta*

**FIGURE 3:**
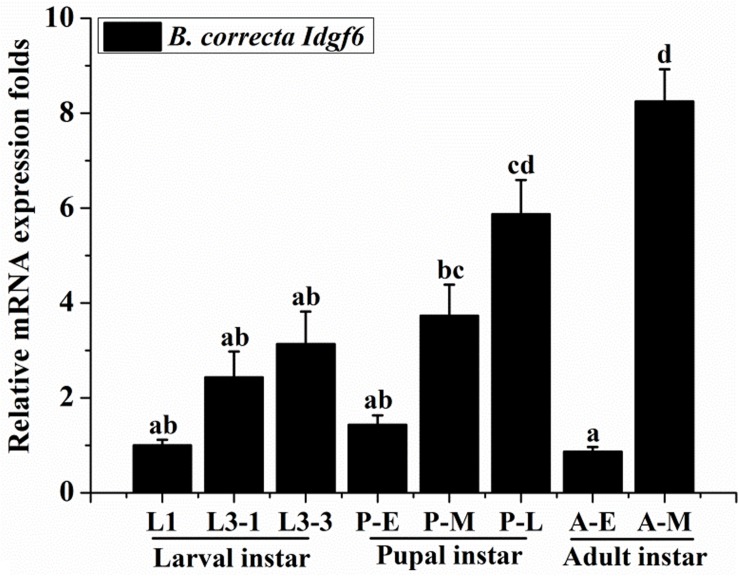
The expression of *Idgf6* at eight developmental stages of *B. correcta*. The eight developmental stages examined include the 1st instar larvae (L1, 2-day-old indicates 2 days post hatching), 3rd early instar larvae (L3-1, 5-day-old), 3rd instar larvae (L3-3, 7-day-old), early pupae (P-E, 1-day-old indicates 1 day post pupating), medium pupae (P-M, 5-day-old), and late pupae (P-L, 9-day-old), early adults (A-E, 1 day post eclosion) and late adults (A-M, 10 days post eclosion). The results are presented as the relative expression after normalization against the endogenous *18S rRNA* gene. Expression is relative to the gene expression in 1^st^ instar larvae (assigned a value of 1), and the same letter means there are no significant differences. Different letters above the bars represent significant differences at *P* < 0.05, as determined by ANOVA followed by Tukey’s HSD tests.

Using qRT-PCR, the expression levels of *Idgf6* differed significantly in certain developmental stages (Tukey HSD tests: *P* < 0.05). In the larval stage, *Idgf6* was expressed in the 1^st^ instar and tended to stabilize until the 3^rd^ instar. In the pupal stage, a lower level of mRNA expression was detected in early pupae, and its expression rose during medium pupae and reached the second highest level in late pupae (*P* = 0.000). In adults, the relative expression of *Idgf6* in late adults was significantly higher than that in early adults, and the highest expression level was measured in late adult individuals (*P* = 0.001). Overall, within each stage, *Idgf6* expression is gradually up-regulated and specially enriched at the late (Figure 3). The different expression levels indicate that *Idgf6* has special physiological roles in different developmental stages.

### Knocking Down *Idgf6* Caused Larval Death and Adult Malformation of *B. correcta*

#### The Functional Study of the Larval Stage

**FIGURE 4:**
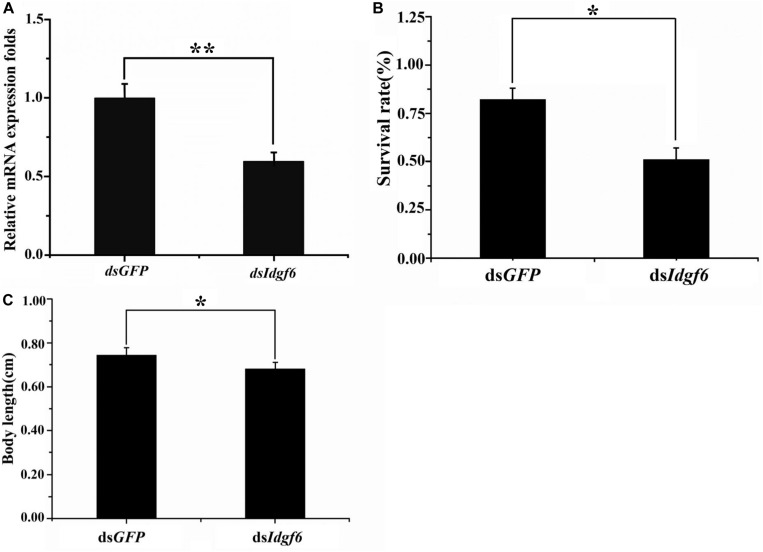
Effect of silencing *Idgf6* in *B. correcta*. **(A)** The relative expression level of *Idgf6* after feeding ds*Idgf6*. **(B)** Larvae survival rate after exposure to dsRNA. **(C)** Larval body sizes after exposure to dsRNA. All the fruit flies in the functional study shown in **Figure 4** were 5-day-old 3rd early instar larvae exposed to dsRNA at a concentration of 1000 ng/μl for 96 h at 25 °C. Five replicates were conducted and the data were presented as mean ± SE. **indicates a statistically significant difference in *Idgf6* mRNA expression between the ds*Idgf6* group and the control ds*GFP* groups (*t*-tests: *P* < 0.01). *indicates a statistically significant difference in survival and body length between the ds*Idgf6* group and the control ds*GFP* group (*t*-test: *P* < 0.05).

**FIGURE 5:**
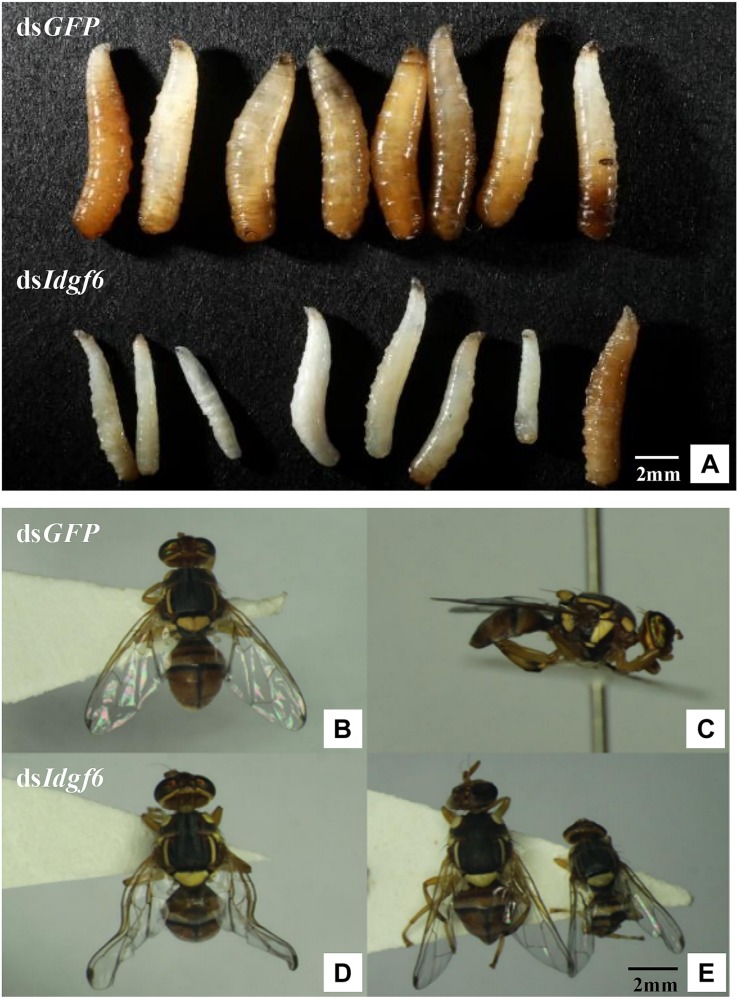
*B. correcta* larval and adult malformed individuals after silencing. **(A)** Malformed phenotype of larvae after feeding ds*GFP* and ds*Idgf6*. **(B)** Front view of the phenotype of adults after feeding ds*GFP*. **(C)** Side view of the phenotype of adults after feeding ds*GFP*. **(D)** First type of malformed phenotype observed in adults after feeding ds*Idgf6*, which resulted in partly extended wings. **(E)** Second type of malformed phenotype of adults after feeding ds*Idgf6*, which led to smaller fruit flies.

After 3^rd^ early instar larvae of *B. correcta* were exposed to ds*Idgf6* at 1000 ng/μl for 96 h, the mRNA expression level of *Idgf6* was significantly reduced by 41.2% (*P* = 0.008) (**Figure 4A**). We also investigated the phenotypic changes of recipient insects after dsRNA treatment. Obvious increases in mortality were observed in treatment groups throughout the feeding period which increased by 37.8% as compared to the control ds*GFP* group (Figure 4B). Then, we measured the body size after feeding. The body sizes of surviving larvae in ds*GFP* and ds*Idgf6* groups were 0.7438 ± 0.1636 and 0.6861 ± 0.1452 after feeding, respectively. Thus, body size for the ds*Idgf6* group decreased by 8.4 % when compared to the control ds*GFP* group (Figures 4C, 5A).

#### The Functional Study of the Pupal Stage

After all the fruit flies emerged, some individuals per treatment were found to exhibit two types of malformation compared with individuals fed ds*GFP* (Figures 5B,C). One type led to smaller body sizes, which accounted for 13.33%, and the other resulted in partly extensible wings, accounting for 6.67%, which led to a loss of flight capacity (Figures 5D,E). During subsequent development, 100% of deformed adults died before sexual maturity, approximately 5 days after emergence. In addition, there were no dead or malformed flies in the control groups, and all the flies lived for more than half a month.

## Discussion

In this study, the full-length cDNA sequence of *Idgf6* was cloned from *B. correcta*. There is one predicted and conserved domain in *B. correcta Idgf6* protein similar to ChiA chitinases, the glycoside hydrolase 18 (GH18) family of chitinases was predicted. In *D. melanogaster*, IDGFs have eight-chain alpha/beta barrel fold associated with GH 18 chitinase, but they have a known amino acid substituent that can eliminate the catalytic activity of chitinase (Kawamura et al., 1999). However, we found there are two amino acid substituents in the Tephritid fruit flies (Figure 1), which may play a similar role in eliminating the catalytic activity of chitinase. The presence of Chi A domain may indicate that *Idgf6* evolved from chitinases and gained new functions as a growth factor with the interaction of cell surface glycoproteins (Kawamura et al., 1999; Varela et al., 2002). Finally, using *Idgf6* nucleotide sequences of Drosophilidae and Tephritidae in GenBank, we applied the maximum-likelihood method to obtain a phylogenetic tree. *Idgf6* in *B. correcta* has high identity with homologues in other Tephritid fruit flies (Figure 2), and *Idgf6* in *B. correcta* has the closest relationship to *B. dorsalis Idgf6*.

*Idgf* genes play an important role in promoting growth, proliferation, cell polarization, and motility (Kawamura et al., 1999). In the present study, an examination of the temporal expression pattern of *Idgf6* revealed that the gene was detectable throughout the development of the insect and highly expressed in the late pupa and adult stages, with lower expression observed in other stages. These results indicate that this gene may play an important role in the whole stage of insect development, mainly in the pupa and adult stages. This result is consistent with previous findings in *D. melanogaster*, in which analysis of DS47 (IDGF6) protein production and mRNA expression during fly development indicated that both are present throughout the entire *D. melanogaster* life cycle, but are relatively lower in the embryos stage (Wang et al., 2009; Pesch et al., 2016). *Idgf6* is expressed in the cuticle producing organs, the mouth hooks, the epidermis, the tracheal system and the posterior spiracles, and especially in larvae, its message is made in the fat body and by hemocytes and secreted into the hemolymph (Kirkpatrick et al., 1995; Pesch et al., 2016). Besides these, it’s very interesting that within each stage, *Idgf6* expression is gradually up-regulated and specially enriched at the late, which may match its special requirement for molting into the next stage (Pesch et al., 2016).

*Idgf6* was depleted at the larval stage, which caused partial larval death and adult wing malformation. However, the percentage of malformation was low, and the potential overlapping function between the six IDGF proteins could contribute to the incomplete penetrance in the RNAi experiments. These experimental results imply that *Idgf6* in *B. correcta* is related to larval mortality and adult wing development. Insect body contains hard, insoluble chitin, which is part of the exoskeleton, trachea, and the peritrophic membrane (PM) that surrounds the midgut food (Merzendorfer and Zimoch, 2003). Chitin is found in a number of different structures in addition to the cuticle and PM (Wilson and Cryan, 1997) whose synthesis occurs throughout insect development, including embryonic, larval, pupal and adult stages (Zhu Q.S. et al., 2004). Chitin in organs provides protection to insects against environmental and mechanical injuries, but it limits the growth and development of insects. Thus, the cuticles and PM are degraded periodically and reshuffled to allow growth and development (Merzendorfer and Zimoch, 2003). Insect chitinase plays a key role in degrading chitin in the old cuticles and PM during the larval molting and purulent process, and chitinase also plays a defensive role to prevent bacteria and fungi from penetrating PM. Therefore, in this study, knocking down the *Idgf6* gene in the larval stage may result in a decrease in the ability to degrade chitin in the old cuticles and a limitation in body size elongation. The decline in defensive ability makes the larvae susceptible to infection by bacteria, fungi, etc., resulting in an increase in larval mortality. According to a previous study, chitin is associated with wing joints in *B. dorsalis*, so it is speculated that the malformation of the adult wing after interference is related to this (Gu et al., 2019).

The GH18 genes of chitinases have potential use for pest management as biopesticides (Kramer and Muthukrishnan, 1997). Homologues of these genes exist in parasites and pests harmful to mankind and agriculture, such as mosquitos, lice and spotted wing *Drosophila* (Zhang et al., 2011b; Eichner et al., 2015; Fan et al., 2015). Insect chitinase genes have been suggested as targets for gene silencing via RNAi and have also been proposed as appropriate candidates in host-mediated silencing of pest genes (HMSPGs) for the control of diseases and insect pests of date palm (Zhu et al., 2008c; Niblett and Bailey, 2012; Al-Ayedh et al., 2016; Su et al., 2016; Cao et al., 2017). Therefore, it is of interest to focus on the functions of the genes involved in development and reproduction for future pest control. The target gene in our study, the *Idgf6* gene, provides a new target for a gene-specific search of pesticides, because the epithelial barrier was damaged and caused premature death of animals, and it is more sensitive to pathogenic infections. Blast searches showed that parts of the N-terminus of *Idgf6* were not conserved among insects, providing a specific target for insecticides. This opens the possibility to search for small molecules that may inhibit the function of insect species-specific *Idgf6* without affecting beneficial organisms. Chitin-ECM is the most important insect barrier against any environmental stresses. Hydrolase enzymes affecting function and the ECM protection system provide new strategies to eliminate already problematic animals in larval stages and prevent the growth of next generation pests (Pesch et al., 2016). Our research implies that silencing the *Idgf6* gene can cause larval death and adult wing malformation. Our findings indicate an essential role of *Idgf6* in larval mortality and adult wing development. Additionally, our results provide new insights into the function of *Idgfs* family members and may reveal a new potential gene for pest control.

## Data Availability Statement

All datasets generated for this study are included in the article/Supplementary Material. The datasets generated for this study can be found in the GenBank accession no. MK450457.

## Author Contributions

YZ, XG, and LL conducted statistical analysis of the whole data. YZ wrote the draft and revised manuscript. YZ, XG, ZL, YS, and LL provided statistical expertise and were involved in data analysis and interpretation of results. LL and ZL conceived and supervised the study. All authors reviewed and made contribution to the final manuscript.

## Conflict of Interest

The authors declare that the research was conducted in the absence of any commercial or financial relationships that could be construed as a potential conflict of interest.

## Footnotes

1 http://www.ncbi.nlm.nih.gov/gorf/gorf.html

2 http://blast.ncbi.nlm.nih.gov/Blast.cgi
