# Supplementary material for: Imaginal Disc Growth Factor 6 (Idgf6) Is Involved in Larval and Adult Wing Development in Bactrocera correcta (Bezzi) (Diptera: Tephritidae)
Source: Front Genet. 2020 May 6;11:451. doi: 10.3389/fgene.2020.00451 (PMC7218075; doi:10.3389/fgene.2020.00451)
Supplement: Supplementary file 1 [file Table_1.docx]

**Supplementary information**

Table S1: **Details of IDGF6 protein sequences used phylogenetic analysis.**

| **No.** | **Species** | **Accession Number** |
| --- | --- | --- |
| 1 | *Bactrocera correcta* | MK_450457 |
| 2 | *Drosophila melanogaster* | NM_057733.4 |
| 3 | *Ceratitis capitata* | XM_004517420.3 |
| 4 | *Drosophila willistoni* | XM_002061003.3 |
| 5 | *Musca domestica* | XM_005181848.3 |
| 6 | *Zeugodacus cucurbitae* | XM_011188848.1 |
| 7 | *Bactrocera dorsalis* | XM_011205683.2 |
| 8 | *Bactrocera oleae* | XM_014244863.1 |
| 9 | *Drosophila suzukii* | XM_017073697.1 |
| 10 | *Drosophila biarmipes* | XM_017112453.1 |
| 11 | *Drosophila rhopaloa* | XM_017133265.1 |
| 12 | *Drosophila takahashii* | XM_017137692.1 |
| 13 | *Drosophila kikkawai* | XM_017169133.1 |
| 14 | *Drosophila ficusphila* | XM_017189010.1 |
| 15 | *Drosophila eugracilis* | XM_017222501.1 |
| 16 | *Drosophila bipectinata* | XM_017252795.1 |
| 17 | *Drosophila elegans* | XM_017269099.1 |
| 18 | *Drosophila miranda* | XM_017293067.1 |
| 19 | *Rhagoletis zephyria* | XM_017632248.1 |
| 20 | *Drosophila busckii* | XM_017982869.1 |
| 21 | *Drosophila navojoa* | XM_018104302.1 |
| 22 | *Bactrocera latifrons* | XM_018946547.1 |
| 23 | *Drosophila serrata* | XM_020952186.1 |
| 24 | *Drosophila obscura* | XM_022369523.1 |
| 25 | *Drosophila hydei* | XM_023308203.1 |
